# Modulation of intestinal IL-37 expression and its impact on the epithelial innate immune response and barrier integrity

**DOI:** 10.3389/fimmu.2023.1261666

**Published:** 2023-09-20

**Authors:** Laura Kröhn, Aline Azabdaftari, Julian Heuberger, Christian Hudert, Matthias Zilbauer, Tilman Breiderhoff, Philip Bufler

**Affiliations:** ^1^ Department of Pediatric Gastroenterology, Nephrology and Metabolic Diseases, Charité Universitätsmedizin Berlin, Berlin, Germany; ^2^ Berlin Institute of Health at Charité - Universitätsmedizin Berlin, Berlin, Germany; ^3^ Department of Hepatology and Gastroenterology, Charité Universitätsmedizin Berlin, Berlin, Germany; ^4^ Wellcome Trust–Medical Research Council Stem Cell Institute, University of Cambridge, Cambridge, United Kingdom

**Keywords:** innate immune response, inflammatory bowel disease, IL-37, intestinal organoid, intestinal barrier, tight junctions, cytokines

## Abstract

**Background and Aims:**

Intestinal epithelial cells separate the luminal flora from lamina propria immune cells and regulate innate immune responses in the gut. An imbalance of the mucosal immune response and disrupted intestinal barrier integrity contribute to the evolution of inflammatory bowel diseases. Interleukin (IL)-37 has broad anti- inflammatory activity and is expressed by the human intestinal epithelium. Mice ectopically expressing human IL-37 show reduced epithelial damage and inflammation after DSS-induced colitis. Here, we investigated the impact of IL-37 on the innate immune response and tight junction protein expression of mouse intestinal organoids and the modulation of *IL37* expression in human intestinal organoids.

**Methods:**

Murine intestinal organoids were generated from IL-37tg and wildtype mice. Human ileal organoids were generated from healthy young donors.

**Results:**

Expression of transgene IL-37 or recombinant IL-37 protein did not significantly reduce overall proinflammatory cytokine mRNA expression in murine intestinal organoids. However, higher *IL37* expression correlated with a reduced proinflammatory cytokine response in murine colonic organoids. *IL37* mRNA expression in human ileal organoids was modulated by proinflammatory cytokines showing an increased expression upon TNF-α-stimulation and decreased expression upon IFN-gamma stimulation. Transgene IL-37 expression did not rescue TNF-α-induced changes in morphology as well as ZO-1, occludin, claudin-2, and E-cadherin expression patterns of murine jejunal organoids.

**Conclusions:**

We speculate that the anti-inflammatory activity of IL-37 in the intestine is mainly mediated by lamina propria immune cells protecting intestinal epithelial integrity.

## Introduction

The intestinal epithelium is the largest epithelial layer in the human body that is in contact with the environment (1). Intestinal epithelial cells (IEC) form the barrier between the intestinal luminal microbiome and the lamina propria immune cell compartment and mediate innate immune responses of the gut (2). Cytokines secreted by either resident or infiltrating immune cells modulate epithelial proliferation, apoptosis, and barrier function, and vice versa, epithelial cells themselves can initiate immune responses at the mucosal level (3). IEC express a variety of cytokine receptors and receptors to sense pathogens or pathogen-associated molecular patterns (2, 4, 5). Subsequent signaling pathways induce cytokine and chemokine secretion by IECs to recruit immune cells to the site of inflammation (2, 4–6) with destructive (7–12) or beneficial effects (13–15) on the intestinal epithelial barrier.

The functional crosstalk between IEC and immune cells via cytokines and chemokines is tightly balanced in the healthy gut (3). In contrast, an impaired gut barrier integrity and imbalance of the mucosal immune response can contribute to inflammatory bowel diseases (16–21). The imbalance of mucosal immune responses may be caused by an exaggerated immune activation or the loss of anti-inflammatory, protective immune mechanisms. As such, it has been shown that, i.e., the overproduction of interleukin (IL)-12 by macrophages (22), genetic variants of NOD2 overactivating NF-κB in monocytes (23), or alterations in TLR expression (20) are associated with Crohn’s disease. On the other hand, reduced anti-inflammatory mechanisms such as disturbed TGF-ß signaling in inflammatory bowel disease (IBD) patients (24), reduced number of regulatory T cells in peripheral blood in patients with ulcerative colitis (25) or IL-10 or IL-10 receptor deficiency in pediatric patients with early-onset enterocolitis (26) are examples of a harmful, overactivated immune response in the gut.

IL-37, an anti-inflammatory member of the IL-1 family, is attributed to balancing the intestinal immune response, and a homozygous loss-of-function variant has been shown to be associated with infantile-onset IBD (27). IL-37 acts via two different pathways. Extracellular IL-37 binds to the IL-18 receptor 1 (IL-18RI) and a single IL-1R-related molecule (SIGIRR), forming the tripartite complex and suppressing MyD88-dependent proinflammatory signals (28, 29). Intracellular IL-37 interacts upon caspase-1 cleavage with Smad3, translocates to the nucleus, and reduces the production of proinflammatory cytokines (30, 31).

IL-37 reduces proinflammatory cytokine secretion in mouse macrophages, human monocytes, and epithelial cells upon stimulation with LPS or IL-1ß (30) and inhibits systemic inflammation in various murine disease models (30, 32–35). IL-37 also displays an anti-inflammatory role within the epithelium, as shown in experiments on T84 colonic carcinoma cells (36), human and murine intestinal organoids (37), and dextran sodium sulfate (DSS)-colitis mice (32). Studies even showed that IL-37 expression was increased in the human intestinal epithelium of IBD patients (38, 39).

Inflammation compromises the integrity of the gut barrier (16, 17, 40). Several proinflammatory cytokines were shown to regulate the expression of tight junction (TJ) proteins, leading to decreased transepithelial electrical resistance (TEER) and increased paracellular permeability (7–12), while anti-inflammatory cytokines protect the intestinal barrier integrity (13–15). The role of IL-37 on the intestinal epithelial proinflammatory response and the intestinal epithelial barrier is not well understood yet.

The aim of this study is to analyze the impact of transgene IL-37 (tgIL-37) on innate immune response and TJ protein expression in murine intestinal organoids and to investigate the regulation of *IL37* expression in human intestinal organoids.

## Materials and methods

### Chemicals and reagents

Reagents were purchased from Sigma-Aldrich GmbH (Munich, Germany) [ethylenediaminetetraacetic acid (EDTA), bovine serum albumin (BSA), sucrose, d-sorbitol], Thermo Fisher Scientific (Munich, Germany) [Dulbecco`s Phosphate - Buffered Solution (DPBS), Hank`s Balanced Salt Solution (HBSS)], Merck Life Science KGaA (Darmstadt, Germany) (Na_2_HPO_4_, KH_2_PO_4_, NaCl, KCl), or other manufacturers as indicated.

### Generation of human organoids

Human intestinal forceps biopsies were taken during routine endoscopies from the terminal ileum of two patients without intestinal inflammation (boy, 16 years; girl, 16 years) at Charité -Universitätsmedizin Berlin and collected in HBSS. Patients and legal guardians gave written consent (EA/134/19).

After sampling, ileum biopsies were incubated in EDTA (2.5 mM), followed by vigorous pipetting to isolate crypts, and then Matrigel was added for organoid culture as described (41). Plates were incubated at 37°C and 7% CO_2_. Organoid medium was prepared as described (**Supplementary Table S1**) and replaced every other day (41). Organoids were passaged every 10 to 14 days.

### Generation of murine organoids, passage, and freezing

C57BL/6J mice expressing human IL-37 (IL-37tg) have been described previously (30). Age-matched mice from the congenic control line (WT) were used as controls. IL-37tg and WT mice were bred under specific pathogen-free conditions. Three 8– 14- week-old mice of each genotype were used for organoid generation.

Jejunal organoids from middle jejunal tissue and colonic organoids from distal colonic tissue were generated as previously described with modifications (42, 43). Gut segments were rinsed with ice-cold DPBS, opened longitudinally, cut into 10-mm pieces, and washed by vortexing several times until the supernatants became clear. Crypts were isolated as described (42) but with an incubation of 20 min for the jejunum and 90 min for the colon in small intestinal or colonic crypt isolation buffer (42). After incubation, biopsies were washed in DPBS. Crypts were then released by vigorously shaking the tissue segments in DPBS, and the supernatant was collected in 0.1% BSA/PBS and centrifuged at 300 ×*g* for 5 min at 4°C. Crypts were diluted to 200 crypts per µl in Matrigel (Corning Limited-Life Sciences, Amsterdam, The Netherlands, 354230) and seeded as 20 µl domes in 48-well plates. Culture plates were incubated for 30 min at 37°C. The respective organoid culture medium (**Supplementary Table S2**) was then added. The medium was supplemented with Y-27632 (Merck Millipore) for the first 2 days. Organoid cultures were maintained at 37°C and 5% CO_2_. The medium was changed every other day, and colonic organoids were passaged every 5–6 days and jejunal organoids every 4–5 days. A detailed description of organoid generation, passage, and freezing is described in the **Supplementary material**.

### Stimulation of murine jejunal and colonic organoids

Jejunal and colonic organoids were thawed as previously described (42), cultured, and passaged at least once before experiments were performed. For experiments, only cultures from passages four to nine were chosen. Jejunal organoids were stimulated with TNF-α (Gibco, Thermo Fisher Scientific) and colonic organoids were stimulated with LPS (O55:B5, Sigma), as indicated in the figure legends.

### Gene expression analysis

Organoids were harvested for mRNA isolation 4 h after stimulation. Culture medium was removed, and Matrigel domes were washed in DPBS. Organoids of four to five domes were resuspended in 1 ml of Trizol (Invitrogen, Carlsbad, CA, USA), incubated at room temperature for 10 min, and vortexed. mRNA was purified from the aqueous phase following the manufacturer’s protocol, including DNAse digestion (GenElute™ Mammalian Total RNA Miniprep Kit, Sigma). Reverse transcription into cDNA was done using 1 µg of total RNA (Applied Biosystems, Carlsbad, CA, USA). Quantitative PCR (qPCR) was performed using the SYBR Green Master Mix (Applied Biosystems). Primer sequences are summarized in **Table 1**. Measurements were quantified using Quant Studio Design and Analysis software (Applied Biosystems). Gene expression was normalized to *Hprt* expression and untreated control condition.

**Table 1:** Gene-specific primer sequences.

Gene	Forward primer	Reverse primer
*Cxcl1*	AGG CTT GCC TTG ACC CTG AA	CAG AAG CCA GCG TTC ACC AG
*Cxcl2*	CTG CCA AGG GTT GAC TTC AA	TTT TGA CCG CCC TTG AGA GT
*Ccl20*	GTG GGT TTC ACA AGA CAG ATG GC	CCA GTT CTG CTT TGG ATC AGC G
*Tnf*	GTG CCT ATG TCT CAG CCT CT	CTG ATG AGA GGG AGG CCAT T
*Tlr4*	AGC TTC TCC AAT TTT TCA GAA CTT C	TGA GAG GTG GTG TAA GCC ATG C
*Tlr5*	TGG ATG GAT GCT GAG TTC CC	TGG CCA TGA AGA TCA CAC CT
*IL37*	TCC TGG ACT CTG GGA ATC TC	AGA GGC TGA GCT CAA GGA TG
*Sigirr*	CAG TGG CTG AAA GAT GGT CTG G	AGT TGA GCA CCA GGA CAC TGG A
*Il18r1*	AGA GCT GAT CCA GGA CAC ATG G	TGG TGG ACA GAA AAC ACG CAG G
*Tjp1*	GTT GGT ACG GTG CCC TGA AAG A	GCT GAC AGG TAG GAC AGA CGA T
*Ocln*	GCA AGT TAA GGG ATC TGC AGA	TCT CCC ACC ATC CTC TTG AT
*Cldn2*	GTC ATC GCC CAT CAG AAG AT	AGG GAA CCA GTC TCC GTT C
*Hprt*	CCT AAG ATG AGC GCA AGT TGA A	ACA GGA CTA GAA CAC CTG CTA A

### Western blotting

For Western blotting, jejunal organoids were harvested 48 h after stimulation. Matrigel domes were dissolved by incubating for 30 min in organoid harvesting solution (Cultrex, R&D systems, Abingdon, UK), centrifuged, and washed in ice-cold DPBS. Pelleted organoids were resuspended in a lysis buffer containing protease inhibitors. The total protein amount was quantified using the bicinchoninic acid assay kit (Thermo Fisher). Each lane of any kD Mini-Protean TGX precast gel was loaded with 17 μg of protein samples and transferred to the PVDF membrane. After blocking (5% milk powder in Tris- buffered saline/0.05% Tween 20), the membrane was stained with mouse monoclonal antibodies against α-claudin-2 (Thermo Fisher; clone 12H12; 1:500), α-occludin (Thermo Fisher; mouse OC-3F10; 1:1,000), α-ZO-1 (Thermo Fisher; clone 1A12; 1:500), and α-actin (Santa Cruz, Biotechnology, Heidelberg, Germany; clone 1A4; 1:1,000). Densitometric analysis was performed with ImageJ, and signal densities were normalized to the signal density of actin.

### Immunofluorescence

For immunofluorescence microscopy, jejunal organoids were harvested with organoid harvesting solution 48 h after stimulation. As previously described (44), 5- μm slides of paraffinized agarose domes containing organoids were generated. After rehydration, antigen retrieval was performed in boiling Tris-EDTA Tween buffer. Slides were incubated in 10% donkey serum/DPBS containing 0.3 % Triton X-100 and with primary antibodies (**Supplementary Table S4**) overnight at 4°C. The next day, slides were stained with secondary antibodies (α-rabbit Alexa488 or α-mouse Alexa647) (Invitrogen; donkey; 1:1,000) for 1 h at room temperature and counterstained with DAPI. Image acquisition was performed with ×25 glycerol immersion on a Zeiss confocal LSM980 NLO microscope with Airyscan, and images were analyzed using Zen software.

### Statistical analysis

Results are expressed as the mean values with 1 standard deviation (SD). A paired Student’s *t*-test was applied to compare treated versus untreated conditions in one experimental group. An unpaired Student’s *t*-test was used for comparisons between organoids generated from WT and IL-37tg mice. For data that were not considered as normally distributed using the Shapiro–Wilk test, the corresponding nonparametric tests (Wilcoxon signed-rank test or Mann–Whitney *U* test) were performed. For correlation analysis, statistical significance was tested with Pearson´s correlation and the Student’s two-tailed *t*-test. Outliers were defined by ROUT (*Q* = 1%) and Grubbs (alpha = 0.05) test. All tests were performed using GraphPad Prism software (version 9.3.1). A *p*-value of 0.05 or less was considered significant.

## Results

### Cytokine response and the expression of IL-37 and its receptor components in WT and tgIL-37 intestinal organoids

**Figure 1:**
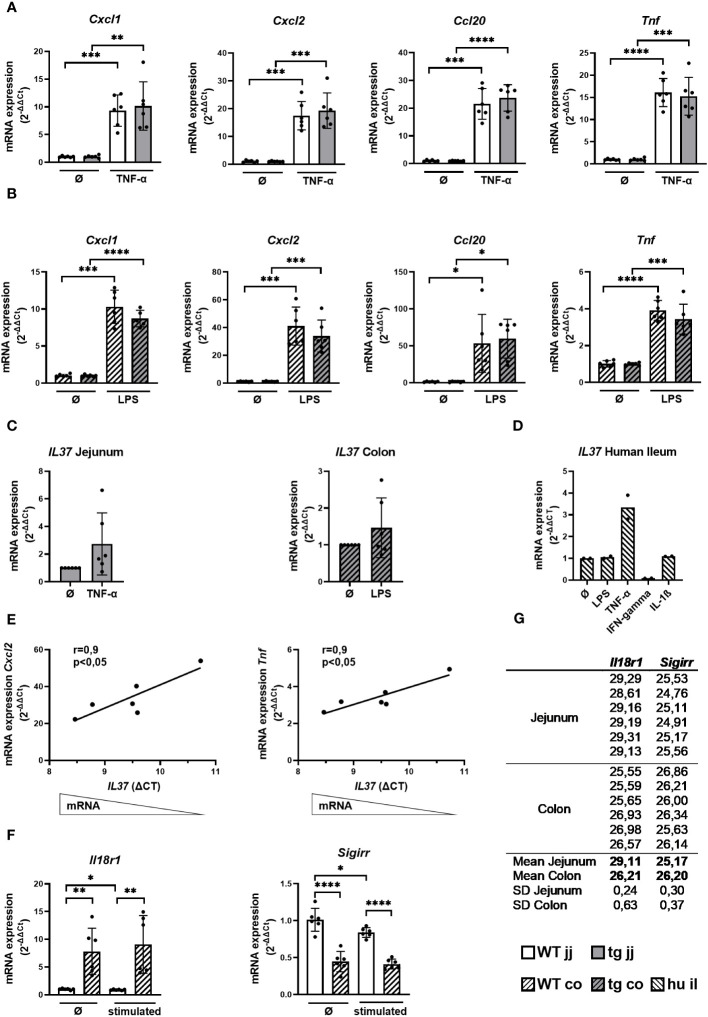
Cytokine response and the expression of IL-37 and its receptor components in WT and tgIL-37 intestinal organoids. Jejunal and colonic organoids derived from IL-37tg and WT mice were stimulated for 4 h with murine TNF-α (10 ng/ml) or LPS O55:B5 (1,000 ng/ml) (37) in culture medium. mRNA expression of cytokines in **(A)** jejunal and **(B)** colonic organoids was measured by qPCR and expressed as fold change relative to the mean of unstimulated organoids of the respective mouse (2^−ΔΔCT^). **(C)** Induction of *IL37* mRNA expression in stimulated IL-37tg-derived organoids was measured by qPCR and expressed as fold change relative to unstimulated organoids (2^−ΔΔCT^). **(D)** Human ileal organoids (il, *n* = 2, two donors) were stimulated for 24 h with LPS O55:B5 (1,000 ng/ml), human TNF-α (10 ng/ml), human IFN-gamma (20 ng/ml), and human IL-1ß (10 ng/ml) in the culture medium. mRNA expression of IL-37 was measured by qPCR and expressed as fold change relative to unstimulated organoids (2^−ΔΔCT^). **(E)** Correlation of cytokine mRNA expression relative to mean of unstimulated colonic organoids of the respective mouse (2^−ΔΔCT^) with *IL37*-ΔCT-values of stimulated colonic organoids derived from IL-37tg mice. **(F)** mRNA expression of *Il18r1* and *Sigirr* in jejunal and colonic WT organoids was measured by qPCR and expressed as fold change relative to mean of unstimulated jejunal organoids (2^−ΔΔCT^). **(G)** Mean CT-values of *Il18r1* and *Sigirr* qPCR duplicates in jejunal and colonic WT organoids. Each experiment was performed with organoid lines of two different mice and repeated three times (in total *n* = 6). Open bars: organoids derived from WT mice (WT); grey bars: organoids derived from IL-37tg mice (tg); no pattern: jejunal organoids (jj); striped pattern: colonic organoids (co). Each data point represents a single organoid line. Data are expressed as the mean with SD. ^*^ *p* ≤ 0.05; ^**^ *p* ≤ 0.01; ^***^ *p* ≤ 0.001; ^****^ *p* ≤ 0.0001.

To corroborate whether *IL37* impacts the immune response of intestinal epithelial cells, we stimulated intestinal organoids generated from IL-37tg and WT mice with TNF-α and IL-1β, as well as LPS and flagellin. Murine jejunal organoids were most potently stimulated by TNF-α and colonic organoids by LPS, inducing the highest mRNA expression levels of *Cxcl1*, *Cxcl2*, *Ccl20*, and *Tnf* within a 4-h stimulation period (**Supplementary Figure S1**; **Figures 1A, B** **)**.

TgIL-37 expression was not associated with a significant change in proinflammatory cytokine mRNA expression in jejunal and colonic organoids before and after stimulation, despite a trend toward reduced *Cxcl1*, *Cxcl2*, and *Tnf* mRNA levels in colonic IL-37tg-derived organoids (**Figures 1A, B** **)**.

Jejunal and colonic organoids generated from IL-37tg mice showed a trend toward higher *IL37* expression after stimulation with TNF-α and LPS, respectively (**Figure 1C**). Similarly, *IL37* expression was threefold induced by TNF-α in human ileal organoids but not by LPS or IL-1β, while IFN-gamma downregulated the expression of *IL37* (**Figure 1D**). *IL37* expression in human ileal organoids was gradually induced by TNF-α over a 24 -h period (**Supplementary Figure S2**).

Baseline *IL37* expression was higher in murine colonic (CT mean: 32.4) versus jejunal organoids (CT mean: 34.4) (**Supplementary Table S5**). In colonic organoids, higher *IL37* mRNA expression was associated with reduced *Cxcl2* and *Tnf* mRNA expression, as indicated by the significant correlation between *IL37-*ΔCt-values of stimulated IL-37tg-derived organoids and their relative *Cxcl2* and *Tnf* mRNA expression (**Figure 1E**).

The lack of correlation between *IL-37* expression and the inflammatory response of jejunal organoids could be due to differences in anti-inflammatory IL-37 signaling. We therefore analyzed the mRNA expression of IL-37 receptor components in jejunal and colonic organoids. *Il18r1* expression was eight times higher in WT colonic organoids compared to WT jejunal organoids before and 10 times higher after stimulation with TNF-α (**Figure 1F**). Baseline *Il18r1* mRNA expression was higher in IL-37tg-derived colonic organoids compared to WT-derived colonic organoids (**Supplementary Figure S3**). *Sigirr* is highly expressed in both jejunal and colonic organoids (CT mean: 25.2 and 26.2) (**Figure 1G**), and the expression was two times higher in WT jejunal organoids compared to WT colonic organoids before and after stimulation (**Figure 1F**). The relation of *Il18r1* and *Sigirr* mRNA expression between jejunal and colonic organoids was similar in IL-37tg organoids and WT organoids (**Supplementary Figure S4**).

### RhIL-37 protein does not modulate the proinflammatory cytokine response in murine jejunal organoids

**Figure 2:**
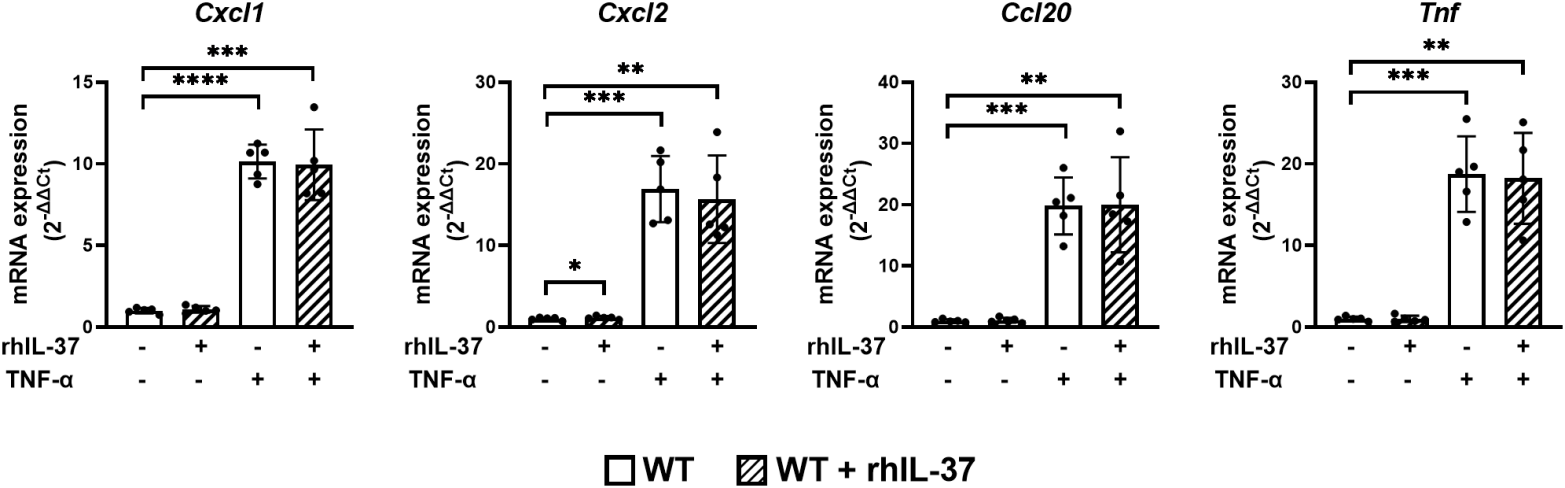
RhIL-37 protein does not modulate the proinflammatory cytokine response in murine jejunal organoids. Jejunal WT-derived organoids were stimulated for 4 h with TNF-α (10 ng/ml) with or without rhIL-37 (100 pg/ml; R&D systems). mRNA expression of cytokines was measured by qPCR and expressed as fold change relative to the mean of unstimulated organoids of the respective mouse (2^−ΔΔCT^). Open bars: organoids derived from WT mice (WT); striped: organoids treated with rhIL-37 (WT + rhIL-37). Each data point represents a single organoid line. Data are expressed as the mean with SD. ^*^ *p* ≤ 0.05; ^**^ *p* ≤ 0.01; ^***^ *p* ≤ 0.001; ^****^ *p* ≤ 0.0001.

IL-37 has intra- and extracellular functionality (28, 30, 31, 45). In order to test the impact of extracellular IL-37 on proinflammatory cytokine expression in gut epithelial cells, we added rhIL-37 to jejunal organoids derived from WT mice (**Figure 2**). rhIL-37 did not modulate the mRNA expression of *Cxcl1*, *Cxcl2*, *Ccl20*, and *Tnf* in jejunal organoids after TNF-α treatment (**Figure 2**).

### Morphologic changes of WT and IL-37tg-derived organoids after TNF-α treatment

**Figure 3:**
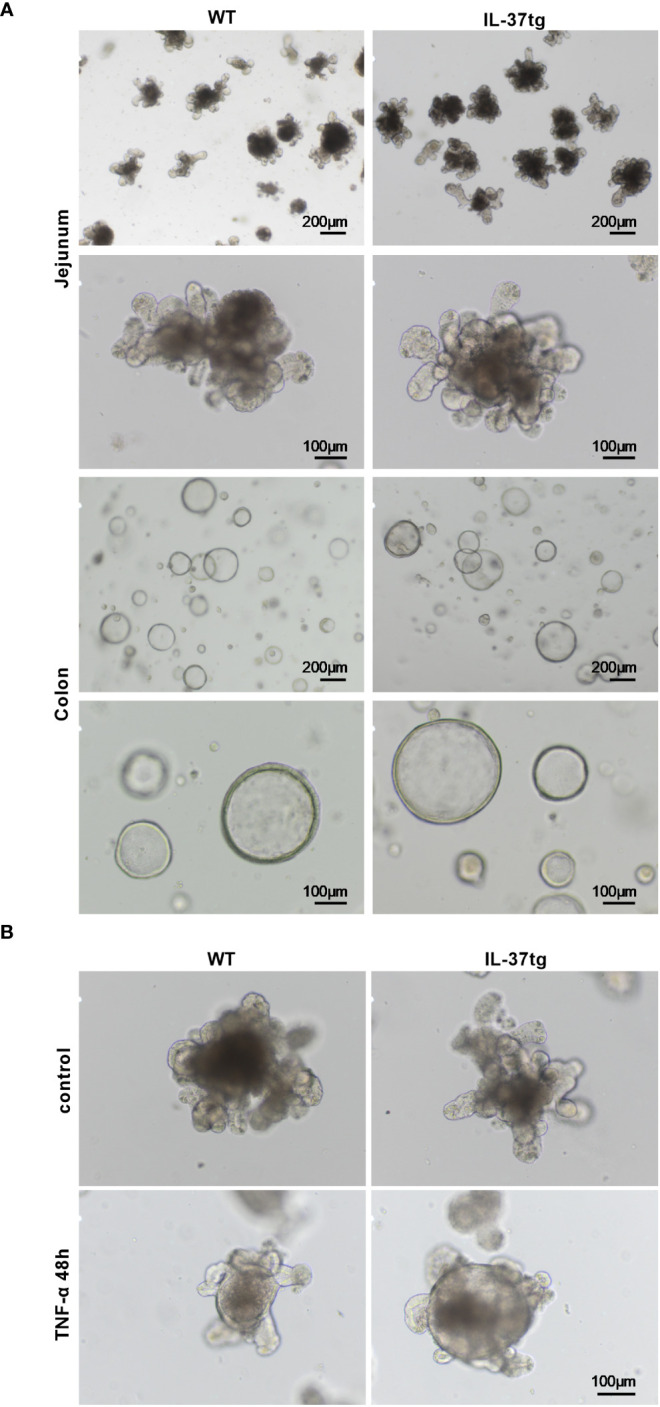
Morphologic changes of WT and IL-37tg-derived organoids after TNF-α treatment. **(A)** Representative light microscope images of murine jejunal and colonic organoids in extracellular matrix on days 3 to 4 under baseline conditions. **(B)** Representative light microscope images of jejunal organoids in extracellular matrix with or without 48 h TNF-α (10 ng/ml) stimulation. One representative picture out of six independent stimulation experiments is shown.

Since *IL37* expression did not markedly alter the intestinal epithelial immune response (**Figures 1A, B**), we evaluated the impact of IL-37 on the morphology of organoids derived from WT and IL-37tg mice during inflammation. Under untreated conditions, the morphology of WT and IL-37tg-derived murine organoids was similar (**Figure 3A**). Jejunal organoids grew as buds and colonic organoids in a spherical shape (**Figure 3A**). After TNF-α treatment, jejunal WT and IL-37tg-derived organoids displayed spherical rounding (**Figure 3B**), while the gross morphology of colonic organoids remained similar after LPS treatment. The shape of organoids was similar in both genetic traits.

### Downregulation and disassembling of intestinal barrier proteins in murine organoids after treatment with inflammatory stimuli are not modulated by tgIL-37 expression

**Figure 4:**
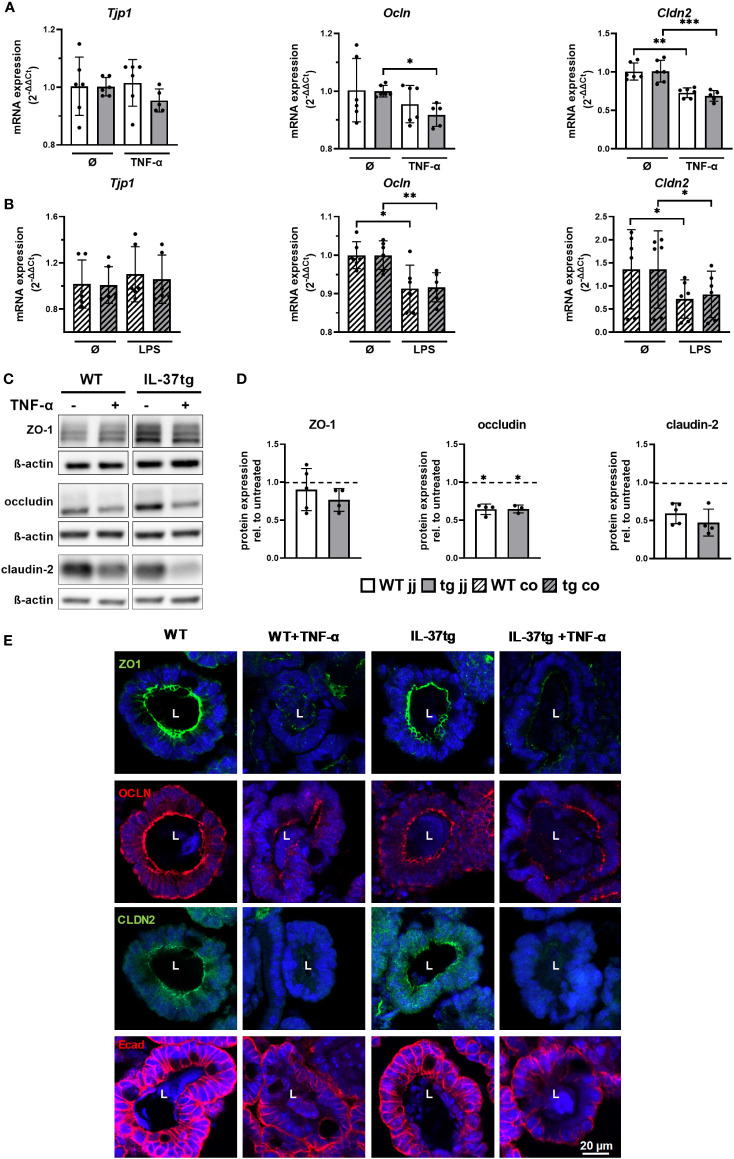
Downregulation and disassembling of intestinal barrier proteins in murine organoids after treatment with inflammatory stimuli is not modulated by tgIL-37 expression. Jejunal and colonic organoids were stimulated for 4 h with TNF-α (10 ng/ml) or LPS (1,000 ng/ml). mRNA expression of TJ proteins ZO-1 (*Tjp1*), occludin (*Ocln*), and claudin-2 (*Cldn2*) in **(A)** jejunal and **(B)** colonic organoids was measured by qPCR and expressed as fold change relative to mean of unstimulated organoids of the respective mouse (2^−ΔΔCT^). For Western blot and immunofluorescence analysis, jejunal organoids were stimulated for 48 h with TNF-α (10 ng/ml). **(C)** Representative Western blots of TJ proteins in jejunal WT- and IL-37tg-derived organoids. **(D)** Densitometric analysis of Western blots of TJ proteins in TNF-α-treated jejunal WT- and IL-37tg-derived organoids. Protein expression is represented as relative to untreated organoids (dotted line). Stars indicate the significance of downregulation after stimulation. **(E)** Representative immunofluorescence images of barrier proteins in WT- and IL-37tg-derived jejunal organoid crypt domains stained with ZO-1 (ZO1, green), occludin (OCLN, red), claudin-2 (CLDN2, green), and E-cadherin (Ecad, red). L, lumens. Open bars: organoids derived from WT mice (WT); closed bars: organoids derived from IL-37tg mice (tg); no pattern: jejunal organoids (jj); striped: colonic organoids (co). Each data point represents a single organoid line. Data are expressed as the mean with SD. ^*^ *p* ≤ 0.05; ^**^ *p* ≤ 0.01; ^***^ *p* ≤ 0.001.

Spherical rounding of small intestinal organoids after TNF-α treatment can be caused by increased TJ permeability (46). Therefore, we analyzed ZO-1, occludin, and claudin-2 mRNA and protein expression in organoids derived from WT and IL-37tg mice before and after respective treatments. There was no significant difference in TJ protein expression between WT- and IL-37-tg-derived organoids on mRNA and protein levels before and after stimulation (**Figures 4A, B, D** **)**. Changes in barrier protein expression and distribution, analyzed by immunofluorescence analysis, were similar in WT- and IL-37tg-derived organoids (**Figure 4E**).

On the mRNA level, *Tjp1* expression, encoding ZO-1, was unchanged after TNF-α treatment in jejunal organoids (**Figure 4A**) and colonic organoids (**Figure 4B**). Expression of *Ocln*, encoding occludin, was reduced by 8% in IL-37tg-derived jejunal organoids (**Figure 4A**), by 9% in WT-derived colonic organoids (**Figure 4B**), and by 8% in IL-37tg-derived colonic organoids (**Figure 4B**). Expression of *Cldn2*, encoding claudin-2, was reduced by 28% in WT-derived jejunal organoids (**Figure 4A**), by 32% in IL-37tg-derived jejunal organoids (**Figure 4A**), by 47% in WT- derived colonic organoids (**Figure 4B**), and by 40% in IL-37tg-derived colonic organoids (**Figure 4B**).

Western blot analysis (**Figures 4C, D** **)** showed a trend toward reduction of ZO-1 in IL-37tg-derived organoids after TNF-α treatment but not in WT organoids. Occludin was reduced by 35% in WT- and by 36% in IL-37tg-derived organoids, and claudin-2 was not significantly reduced by 43% in WT- and by 51% in IL-37tg-derived organoids. Claudin-2 baseline expression is highly varied between single organoid lines, independent of the genotype (**Supplementary Figure S5**).

In the immunofluorescence analysis (**Figure 4E**), ZO-1, occludin, and claudin-2 displayed a strong signal at the apical side of epithelial cells, indicating expression within the TJs. Additionally, occludin showed basolateral staining and claudin-2 an intracellular staining. E-cadherin was highly enriched at the basolateral side and at the lateral membranes, indicating expression within the adhering junctions. ZO-1, occludin, and claudin-2 expression patterns were largely disrupted by TNF-α treatment. E-cadherin staining was restricted to the basolateral side and less detectable at the lateral membranes and at the apical sides after TNF-α treatment. Other than ZO-1, occludin, and E-cadherin, claudin-2 was not expressed homogenously within the organoids but was predominately located within crypt domains (**Supplementary Figure S6**).

## Discussion

In this study, we show that IL-37 does not modulate the gross cytokine response of murine intestinal organoids after immune stimulation with TNF-α or LPS by using tgIL-37 mice or treatment by rhIL37. However, we show that *IL37* expression is upregulated upon proinflammatory stimulation in murine and human organoids. More subtle analyses demonstrate a negative correlation between *IL37* expression and proinflammatory cytokine response in murine colonic organoids. IL-37 did not alter the TNF-dependent downregulation and structural alteration of TJ proteins in murine jejunal organoids.

There was no significant change in proinflammatory cytokine mRNA expression by IL-37tg jejunal or colonic organoids after TNF-α or LPS stimulation. In accordance with these results, we could also not observe differences in cytokine response after treatment with rhIL-37. This indicates that IL-37, expressed within the intestinal epithelium, does not grossly limit the immune response of IEC in an auto- or paracrine manner. Differences within the experimental approach might explain the contrasting results to the observation of Allaire and colleagues describing a downregulation of proinflammatory *Ccl20* and *Cxcl2* in flagellin- and IL-1ß-stimulated murine colonic organoids by treatment with rhIL-37 protein (37). Accordingly, Günaltay et al. showed that CRISPR/Cas knockdown of *IL37* increases the spontaneous chemokine expression in T84 IECs (36). We previously reported that hematopoietic-derived IL-37 from bone marrow transplantation is sufficient to ameliorate the severity of DSS-induced colitis in WT mice (32). This indicated the relevance of tgIL-37 expressed by immune cells to control mucosal inflammation. Since neither tgIL-37 nor rhIL-37 protein modulated the epithelial immune response in our organoid model, we speculate that predominantly extracellular IL-37, released from IECs at the site of tissue inflammation, contributes to the control of the immune response of intestinal mucosa-associated immune cells.

*IL37* expression in IL-37tg mice is driven by a constitutively active CMV-promotor (30). However, like in healthy human tissue, baseline levels of IL-37 mRNA and protein are low in unstimulated IL-37tg mice, which is due to mRNA instability elements within the coding region of IL-37 (30, 47). *IL37* expression is also low in resting jejunal and colonic organoids generated from IL-37tg mice, but we were able to show a trend toward increased levels after stimulation. In human ileal organoids, only TNF-α but not LPS or IL-1ß induced *IL37* expression, while IFN-gamma reduced *IL37* expression. This is consistent with observations on T84 epithelial colon carcinoma cells (38). Upregulation of *IL37* by TNF-α is also in line with a high epithelial *IL37* expression in IBD correlating with higher disease activity (38, 48). In murine and human small intestinal organoids, there was only a minor inflammatory response to LPS, which might be caused by low expression of TLR4 in both and missing TLR4-associated accessory protein expression in human organoids [**Supplementary Figure S1** and as previously shown (49)]. Insufficient immune stimulation could therefore explain the lack of *IL37* induction after LPS treatment in human ileal organoids. The induction of *IL37* by TNF-α in human ileal organoids was similar to that in murine jejunal organoids, corresponding to a high immune stimulation by TNF-α in murine and may be as well in human small intestinal organoids (**Supplementary Figure S1**).

Increasing levels of tgIL-37 mRNA, induced by proinflammatory signals, correlated with a lower *Cxcl2* and *Tnf* response in LPS-stimulated colonic organoids. This negative correlation was not observed in jejunal organoids after stimulation. Since SIGIRR is evenly expressed throughout the murine gut (50), as similarly seen in our jejunal and colonic organoids, we speculate that a higher expression of the IL-37 receptor component *Il18r1* (51–53) in colonic organoids sensitizes for autologous, anti-inflammatory IL-37 signaling. Indeed, polymorphisms in the IL18RI-IL18RAP locus are associated with adult and early-onset IBD (54–56), underlining the relevance of the IL-18RI for human disease activity to target both proinflammatory IL-18 and anti-inflammatory IL-37 immune pathways. In addition, tgIL-37 expression itself is associated with a markedly higher *Il18r1* expression in our IL-37tg colonic organoids, suggesting a regulatory effect of IL-37 on bona fide *Il18r1* expression (**Supplementary Figure S3**). Pretreatment with rhIL-37 was shown to induce SIGIRR expression in humans, LPS-stimulated PBMCs and M1 macrophages (45) indicating that IL-37 induces upregulation of its receptor components to increase its anti-inflammatory capacity.

TJ proteins claudin-2, ZO-1, or occludin are key determinants of epithelial integrity against mucosal inflammation in IBD (17, 18, 40, 57). The impact of TNF-α on epithelial barrier function was extensively studied in 2D cell culture models (10–12, 58). 3D organoid models are a good representation of the epithelial architecture, with a villi-like structure and composition of a variety of epithelial subtypes. However, studies of TJ proteins and IL-37 in organoids are scarce. In our model, TNF-α and LPS caused significant changes in TJ protein expression and morphology in jejunal and colonic organoids. Nevertheless, overall TJ protein expression and organoid morphology were similar between WT- and IL-37tg-derived organoids before and after stimulation. This indicates that tgIL-37 expression in IEC does not prevent the disturbance of TJ protein expression during inflammation.

In summary, our 3D intestinal organoid model enabled us to investigate the impact of IL-37 on IEC innate immune responses and intestinal barrier function, irrespective of epithelial immune cell activation. We show that *IL37* expression is induced in intestinal epithelial cells but has no major impact on modulating IEC innate immune responses itself and does not prevent murine intestinal organoids from altered barrier protein expression after stimulation. We conclude that the protective effect of IEC-derived IL-37 on the intestinal epithelial barrier in murine models of IBD and potentially the human gut is more mediated by downregulating the release of proinflammatory mediators from lamina propria immune cells than intestinal epithelial cells.

## Data availability statement

The raw data supporting the conclusions of this article will be made available by the authors, without undue reservation.

## Ethics statement

The studies involving humans were approved by Ethics committee Charité Universitätsmedizin Berlin. The studies were conducted in accordance with the local legislation and institutional requirements. Written informed consent for participation in this study was provided by the participants’ legal guardians/next of kin. The animal study was approved by Landesamt für Gesundheit und Soziales Berlin. The study was conducted in accordance with the local legislation and institutional requirements.

## Author contributions

LK: Conceptualization, Writing – review & editing, Data curation, Formal Analysis, Investigation, Methodology, Visualization, Writing – original draft. AA: Writing – original draft, Conceptualization, Data curation, Investigation, Methodology, Supervision, Writing – review & editing. JH: Methodology, Supervision, Writing – review & editing. CH: Writing – review & editing, Resources. MZ: Conceptualization, Resources, Methodology, Supervision, Writing – review & editing. TB: Project administration, Writing – original draft, Conceptualization, Investigation, Methodology, Supervision, Writing – review & editing. PB: Writing – review & editing, Writing – original draft, Data curation, Resources, Visualization, Conceptualization, Funding acquisition, Investigation, Methodology, Project administration, Supervision, Validation.
