# Supplementary material for: Modulation of intestinal IL-37 expression and its impact on the epithelial innate immune response and barrier integrity
Source: Front Immunol. 2023 Sep 20;14:1261666. doi: 10.3389/fimmu.2023.1261666 (PMC10548260; doi:10.3389/fimmu.2023.1261666)
Supplement: Supplementary file 1 [file DataSheet_1.docx]

Supplementary Material

# Supplementary Tables

**Supplementary table 1.** Human organoid media components.

| Reagent | Final concentration |
| --- | --- |
| ADF+++ (Table 2) | 30 % (v/v) |
| WRN-CM | 50 % (v/v) |
| Wnt3a-CM | 20 % (v/v) |
| Nicotinamide (Sigma) | 10 mM |
| N-Acetylcysteine (Sigma) | 1.25 mM |
| mEGF (Invitrogen) | 50 ng/ml |
| A3801 (Tocris) | 500 nM |
| SB202190 (Sigma) | 10 μM |
| Primocin (Invitrogen) | 500 μg/ml |
| B-27 Supplement (Invitrogen) | 1 X |

**Supplementary table 2.** Murine organoid media components.

| Reagent | Jejunum  (ENR) | Colon  (WENR) |
| --- | --- | --- |
| ADF+++ (Table 3) | 75 % (v/v) | 50 % (v/v) |
| R-spo-CM | 25 % (v/v) | - |
| L-WRN-CM | - | 50 % |
| mEGF (Invitrogen) | 50 ng/ml | 50 ng/ml |
| mNoggin (Preprotech) | 100 ng/ml | - |
| B-27 Supplement (Invitrogen) | 1 X | 1 X |
| N-Acetylcysteine (Sigma) | 1 mM | 1,25 mM |

Note: L-WRN conditioned medium (CM) was produced from stable transfected adherent fibroblast cells (ATCC) with Wnt-3A, R-spondin and Noggin as described in (1). Wnt3a-CM was produced from stable transfected fibroblasts (ATCC) with Wnt-3A as described in (2). R-spo-CM was produced from stable transfected HEK293 cells (Clinical Physiology Charité Berlin) with R-spondin according to (3).

**Supplementary table 3.** ADF+++.

| Reagent | Final concentration |
| --- | --- |
| Advanced DMEM/F12 (ADF) (Invitrogen) | 1 X |
| GlutaMax (Invitrogen) | 2 mM |
| HEPES buffer (Sigma) | 10 mM |
| Penicillin/Streptomycin (Invitrogen) | 0,5 U/ml |

**Supplementary table 4. Primary immunofluorescence antibodies.**

| Antibody | Animal Species | Concentration | Company |
| --- | --- | --- | --- |
| Ki-67 | rabbit D3B5 | 1:200 | cell signaling (Danvers, USA) |
| E-Cadherin | mouse 36 | 1:200 | BD (Heidelberg, Germany) |
| ZO-1 | mouse 1A12 | 1:200 | Thermo Fisher Scientific (Munich, Germany) |
| claudin-2 | rabbit MH44 | 1:200 | Thermo Fisher Scientific |
| occludin | mouse OC-3F10 | 1:200 | Thermo Fisher Scientific |
| IL-37 | rabbit | 1:80 | Sigma-Aldrich GmbH (Munich, Germany) |

**Supplementary table 5. Baseline *IL37* expression in murine jejunal and colonic organoids.**

|  | *IL37* (CT mean) |  | *IL37* (ΔCT mean) |
| --- | --- | --- | --- |
| Jejunum IL37-tg 1.1 | 32,63 |  | 12,81 |
| IL37-tg 1.2 | 33,97 |  | 14,14 |
| IL37-tg 1.3 | 35,70 |  | 15,46 |
| IL37-tg 2.1 | 34,58 |  | 14,76 |
| IL37-tg 2.2 | 34,47 |  | 14,63 |
| IL37-tg 2.3 | 35,29 |  | 15,05 |
| Colon IL37-tg 1.1 | 32,00 |  | 9,14 |
| IL37-tg 1.2 | 32,81 |  | 10,67 |
| IL37-tg 1.3 | 34,23 |  | 10,57 |
| IL37-tg 2.1 | 30,59 |  | 8,41 |
| IL37-tg 2.2 | 32,05 |  | 9,88 |
| IL37-tg 2.3 | 32,89 |  | 10,24 |
| Mean Jejunum | **34,44** |  | **14,47** |
| Mean Colon | **32,43** |  | **9,82** |
| SD Jejunum | 0,98 |  | 0,85 |
| SD Colon | 1,10 |  | 0,81 |

# Supplementary Figure legends

**Supplementary Figure 1.** Evaluation of best proinflammatory stimulus in jejunal and colonic murine organoids. Jejunal and colonic organoids were stimulated for 4 h with TNF-α (10 ng/ml), IL-1ß (10 ng/ml), LPS (1000 ng/ml) and Flagellin (100 ng/ml) in the culture medium. mRNA expression of cytokines was measured by qPCR and expressed as fold change relative to unstimulated organoids (2^-ΔΔCT^). No pattern: jejunal organoids (jj), striped: colonic organoids (co).

**Supplementary Figure 2.** *IL37* expression over 24 hours in human ileal organoids. Human ileal organoids were stimulated for 6, 12 and 24 h with TNF-α (10 ng/ml). mRNA expression of IL-37 was measured by qPCR and expressed as fold change relative to unstimulated organoids (2^-ΔΔCT^). Each data point represents a single organoid line.

**Supplementary Figure 3.** *Il18R1* mRNA is higher expressed in colonic IL-37tg-derived organoids than WT organoids. mRNA expression of *Il18r1* in colonic WT- and IL-37tg-derived organoids was measured by qPCR and expressed as ΔCT-values. Please note: The lower the ΔCT-value, the higher the IL-37 mRNA expression. Open bars: organoids derived from WT mice, closed bars: organoids derived from IL-37tg mice. Each data point represents a single organoid line. Data are expressed as the mean with SD. *p ≤ 0.05, **p ≤ 0.01, ***p ≤ 0.001, **** p ≤ 0.0001.

**Supplementary Figure 4.** mRNA expression of *Il18r1* and *Sigirr* in murine jejunal and colonic organoids. mRNA expression was measured by qPCR and expressed as fold change relative to mean of unstimulated jejunal organoids (2^-ΔΔCT^). Open bars: organoids derived from WT mice (WT), closed bars: organoids derived from IL-37tg mice (tg), no pattern: jejunal organoids (jj), striped: colonic organoids (co). Each data point represents a single organoid line. Data are expressed as the mean with SD. *p ≤ 0.05, **p ≤ 0.01, ***p ≤ 0.001, **** p ≤ 0.0001.

**Supplementary Figure 5.** Western blots of TJ proteins in WT and IL-37tg-derived jejunal organoids. Jejunal organoids were stimulated for 48 h with TNF-α (10 ng/ml) in the culture medium. Experiments were performed for 3 times (methodical control) with organoid lines of 2 different mice per experimental group (WT1, WT2, IL-37tg1, IL-37tg2) (biological controls). Outliers, identified by Grubbs test of densitometric analysis values have been removed for statistical tests. Protein size is indicated in kDa. Please note the high variation of baseline claudin-2 expression in single organoid lines, that is independently of the murine genotype (WT or IL-37tg).

**Supplementary Figure 6.** Claudin-2 is predominantly expressed in crypt domains of jejunal organoids. Representative immunofluorescence staining of claudin-2 (green) in a single jejunal organoid. Frames indicate crypt domains of organoid. L indicates lumens.

# Supplementary Methods

## Generation of murine organoids, passage and freezing

Jejunal organoids from middle jejunal tissue and colonic organoids from distal colonic tissue have been generated as previously described with modifications (4,5). Gut segments were rinsed with ice-cold DPBS, opened longitudinally, cut into 10-mm-pieces and washed by vortexing several times until supernatants became clear. Crypts were isolated as described (4), but with an incubation of 20 min for jejunum and 90 min for colon in small intestinal or colonic crypt isolation buffer respectively. After incubation biopsies were washed in DPBS. Then, crypts were released by vigorously shaking the tissue segments in DPBS, supernatant was collected in a 50-ml-falcon, filled with 2 ml 0.1 % BSA/PBS and 2 ml HBSS (1X) + CaCl_2_ and MgCl_2_. The procedure was repeated for 5 times, collecting each supernatant in a separate tube as fractions 1 to 5. Each fraction was evaluated under the microscope, fractions enriched with crypts and little cell debris were chosen, filtered through a 70 µm filter, collected in one 50 ml falcon and centrifuged at 300 x g for 5 min at 4°C. Pellet was diluted in Advanced DMEM/F12 (Invitrogen) and the desired amount of crypts was transferred to an Eppendorf tube and centrifuged at 1200 x g for 3,5 min. Then, crypts were diluted to 200 crypts per µl in Matrigel (Corning, 354230) and seeded as 20 µl domes on a 48-well-plate. Culture plates were incubated for 30 min at 37°C. Then, respective organoid culture medium was added. The medium was supplemented with Y-27632 (Millipore) for the first 2 days. Organoid cultures were maintained at 37°C and 5% CO_2_. Medium was changed every other day and colonic organoids were passaged every 5-6 days and jejunal organoids every 4-5 days.

For passage, at most 8 Matrigel domes were collected in 1.5 ml tubes together with the old culture medium. Jejunal organoids were transferred to a 1.5 ml tube and disrupted into fragments by pipetting 3-5 times through a 26-G-cannula, washed in ice-cold ADF+++, centrifuged at 1200 x g for 3.5 min and seeded in Matrigel as described before. Colonic organoids were transferred to a BSA-coated 15-ml Falcon, washed in ice-cold ADF+++, centrifuged at 500 x g for 5 min, resuspended in 500 µl phenol red-free TrypLE (Gibco) and incubated for 5 min at 37°C. Then they were separated into single cells by pipetting 10-14 times with p1000 tip, washed again in ice-cold ADF+++, centrifuged and seeded in a ratio 200 cells/µl Matrigel. After passage, culture medium supplemented with Y-27632 was added for the first two days.

Organoids were frozen after 1 to 10 passages. Jejunal organoids were frozen at day 1 after passage as organoid fragments. Old medium was removed, Matrigel domes were dissolved using Organoid Harvesting Solution (Cultrex) following the manufacturer’s instructions with an incubation time of 30 min and organoid fragments of maximal 10 wells were resuspended in 500 µl Recovery Cell Culture Freezing Medium (Gibco). Colonic organoids were frozen as single cells directly after passage. Therefore, single cells of maximal 10 wells were resuspended after second washing in 500 µl Recovery Cell Culture Freezing Medium (Gibco). Jejunal and colonic organoids were transferred to a cryovial, stored overnight in a Freezing Container with a cooling rate -1 °C/min at -80 °C and were transferred to liquid nitrogen the next day.

1. Miyoshi H, Stappenbeck TS. In vitro expansion and genetic modification of gastrointestinal stem cells in spheroid culture. *Nat Protoc* (2013) 8:2471–2482. doi: 10.1038/nprot.2013.153

2. Fujii M, Matano M, Nanki K, Sato T. Efficient genetic engineering of human intestinal organoids using electroporation. *Nat Protoc* (2015) 10:1474–1485. doi: 10.1038/nprot.2015.088

3. Kim K-A, Kakitani M, Zhao J, Oshima T, Tang T, Binnerts M, Liu Y, Boyle B, Park E, Emtage P, et al. Mitogenic Influence of Human R-Spondin1 on the Intestinal Epithelium. *Science* (2005) 309:1256–1259. doi: 10.1126/science.1112521

4. Mizutani T, Clevers H. “Primary Intestinal Epithelial Organoid Culture.,” In: Ordóñez-Morán P, editor. *Intestinal Stem Cells: Methods and Protocols*. Methods in Molecular Biology. New York, NY: Springer US (2020). p. 185–200 doi: 10.1007/978-1-0716-0747-3_11

5. Grinat J, Kosel F, Goveas N, Kranz A, Alexopoulou D, Rajewsky K, Sigal M, Stewart AF, Heuberger J. Epigenetic modifier balances Mapk and Wnt signalling in differentiation of goblet and Paneth cells. *Life Sci Alliance* (2022) 5:e202101187. doi: 10.26508/lsa.202101187
