# Supplementary figures and images for: Modulation of intestinal IL-37 expression and its impact on the epithelial innate immune response and barrier integrity
Source: Front Immunol. 2023 Sep 20;14:1261666. doi: 10.3389/fimmu.2023.1261666 (PMC10548260; doi:10.3389/fimmu.2023.1261666)

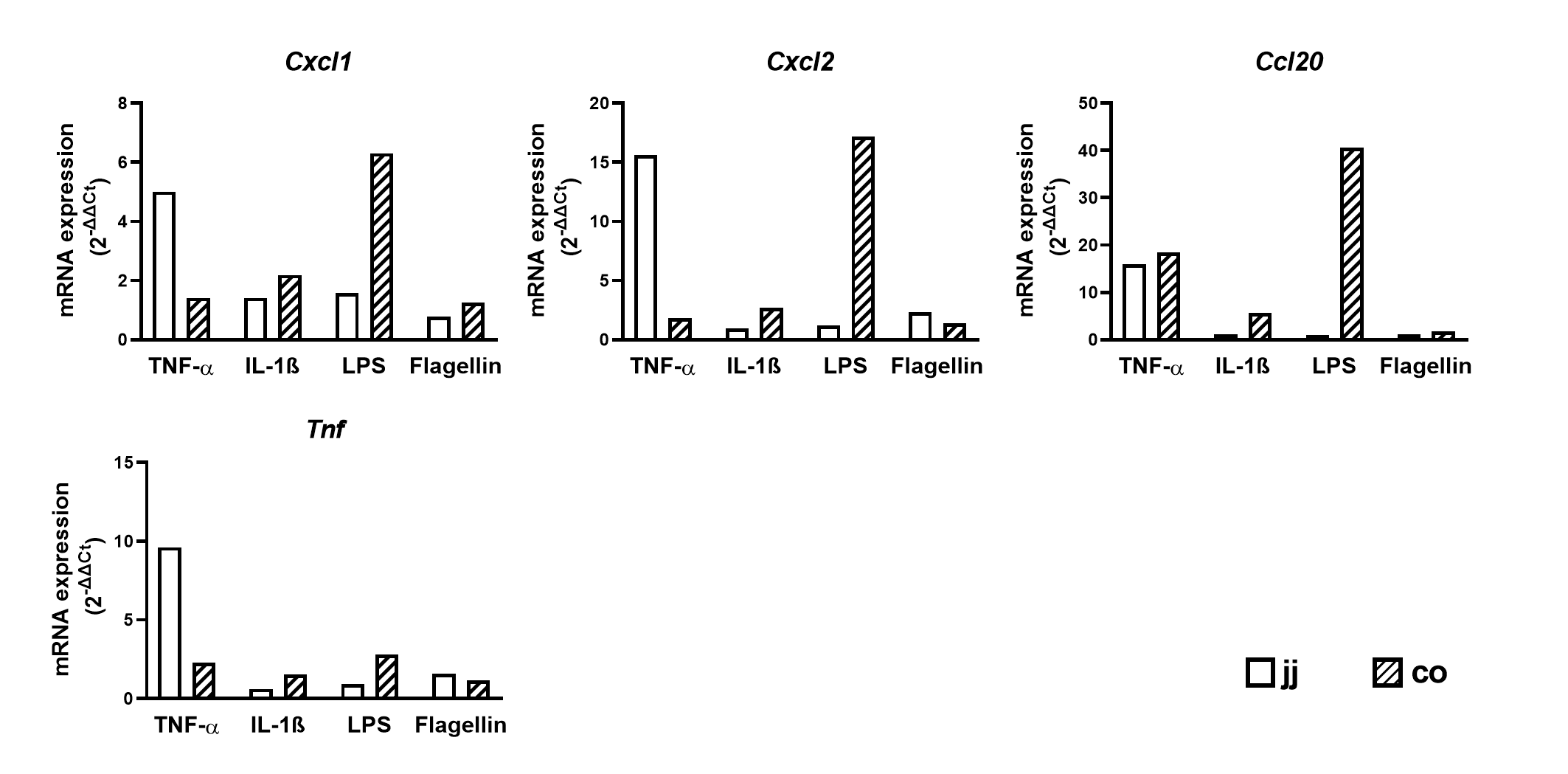

Supplement: Supplementary file 2 [file Image_1.tif]

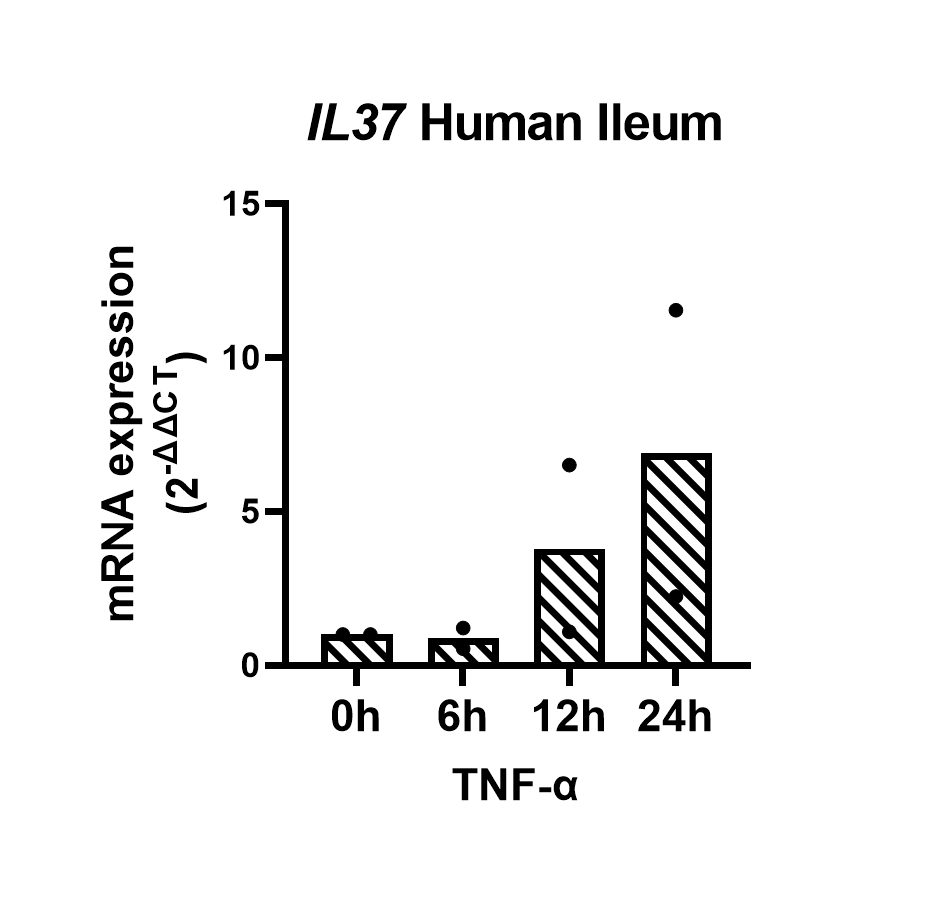

Supplement: Supplementary file 3 [file Image_2.tif]

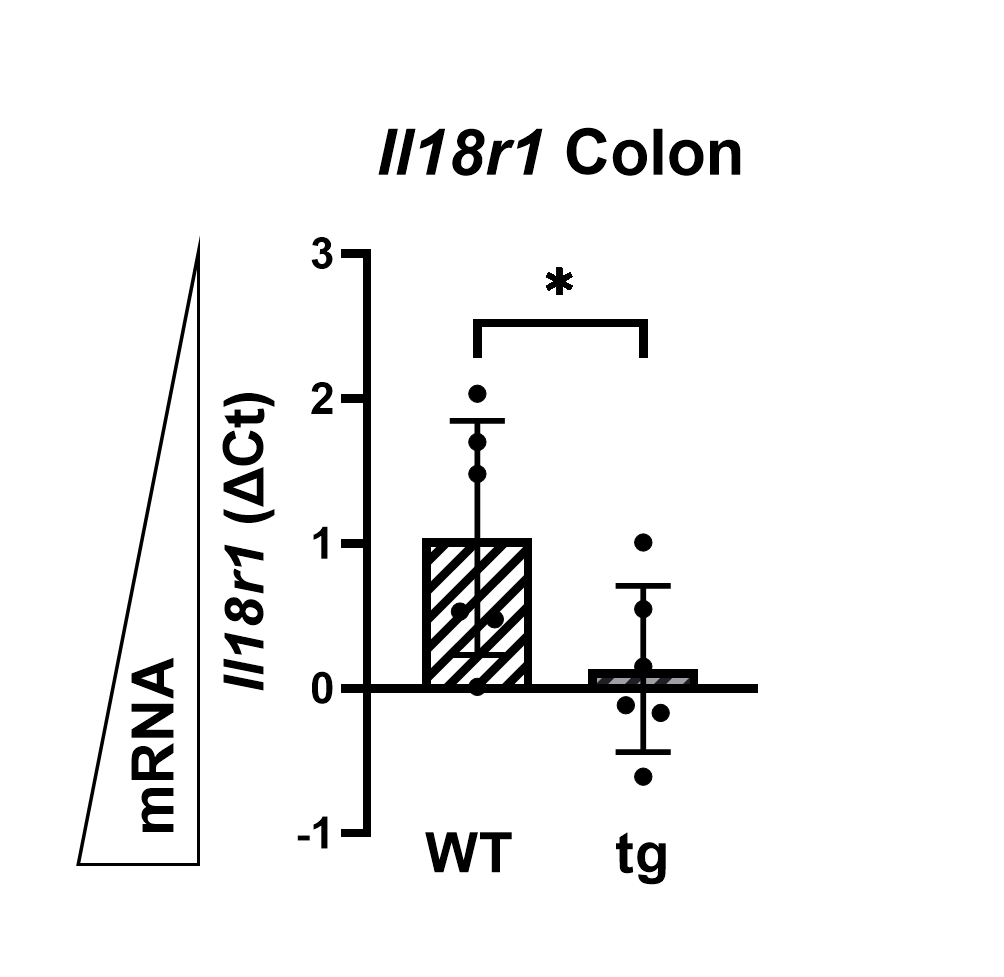

Supplement: Supplementary file 4 [file Image_3.tif]

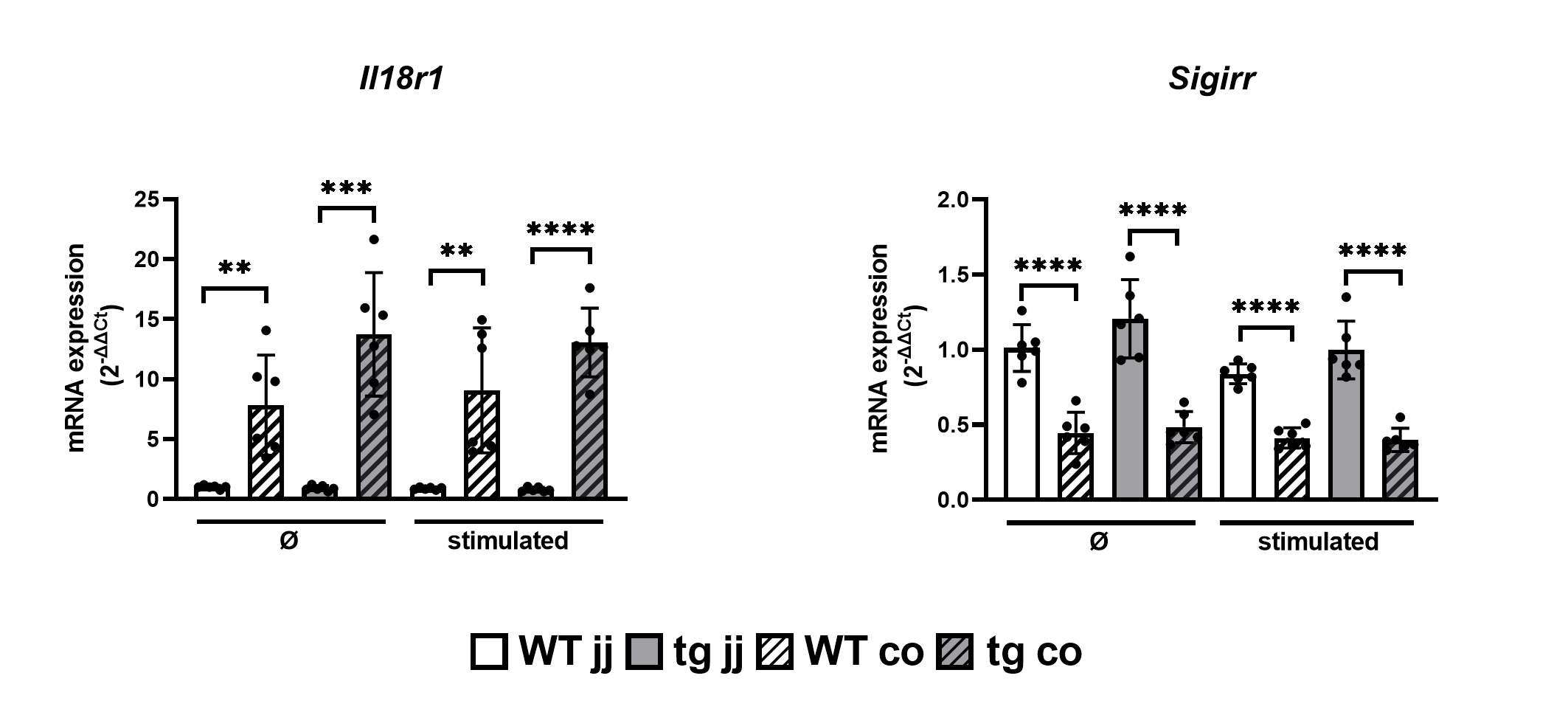

Supplement: Supplementary file 5 [file Image_4.tif]

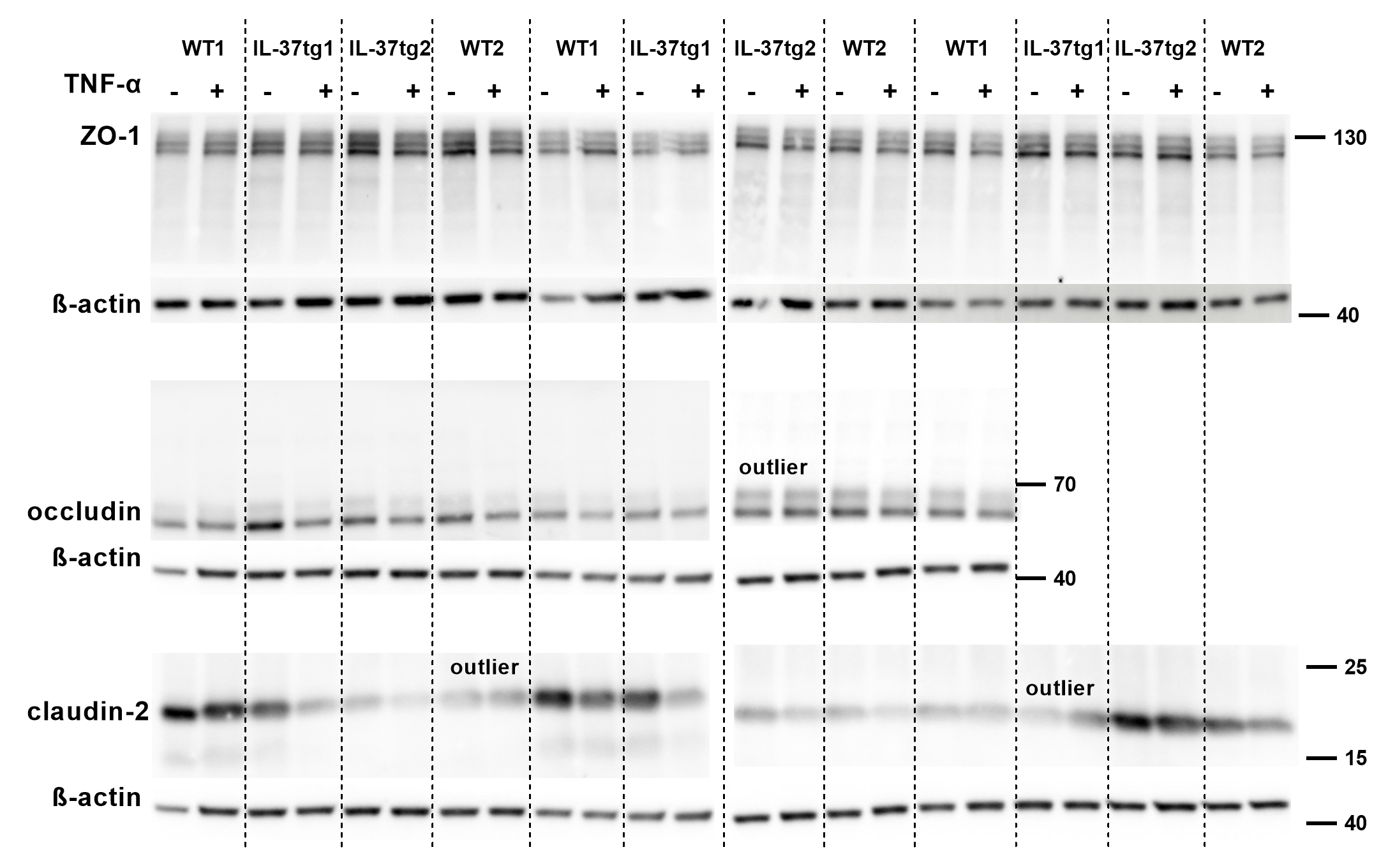

Supplement: Supplementary file 6 [file Image_5.tif]

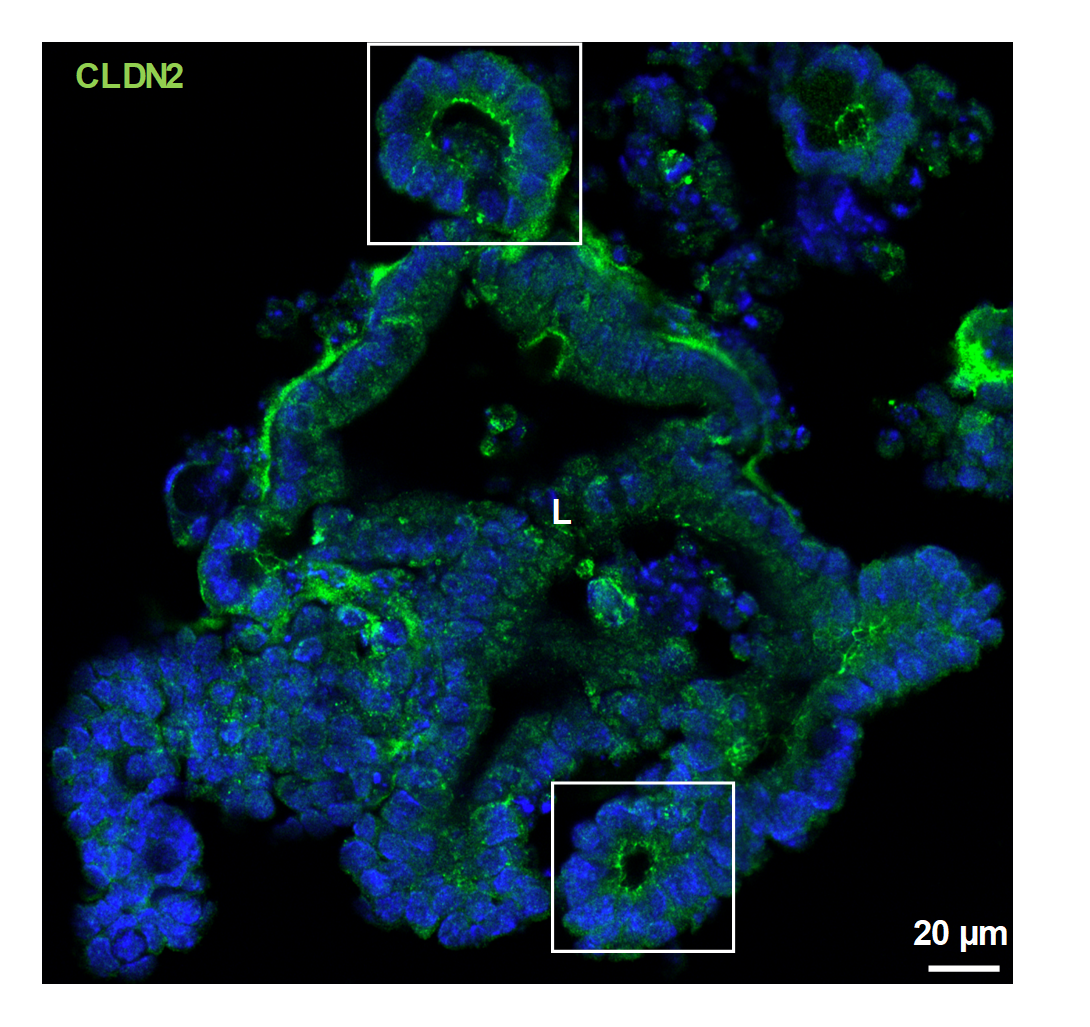

Supplement: Supplementary file 7 [file Image_6.tif]
